# Risk factors and outcome of Chimeric Antigen Receptor T-Cell patients admitted to Pediatric Intensive Care Unit: CART-PICU study

**DOI:** 10.3389/fimmu.2023.1219289

**Published:** 2023-08-02

**Authors:** Marina Caballero-Bellón, Anna Alonso-Saladrigues, Sara Bobillo-Perez, Anna Faura, Laura Arqués, Cristina Rivera, Albert Català, Jose Luis Dapena, Susana Rives, Iolanda Jordan

**Affiliations:** ^1^ Department of Hematology/Oncology, Pediatric Cancer Center Barcelona, Hospital Sant Joan de Déu University of Barcelona, Barcelona, Spain; ^2^ Paediatric Intensive Care Unit, Hospital Sant Joan de Déu, University of Barcelona, Barcelona, Spain; ^3^ Immunological and Respiratory Disorders in the Paediatric Critical Patient Research Group, Institut de Recerca Sant Joan de Déu, University of Barcelona, Barcelona, Spain; ^4^ Centro de Investigación Biomédica en Red de Enfermedades Raras (CIBERER), Instituto de Salud Carlos III, Madrid, Spain; ^5^ Paediatric Infectious Diseases Research Group, Institut de Recerca Sant Joan de Déu, Centro de Investigación Biomédica en Red de Epidemiología y Salud Pública (CIBERESP), Barcelona, Spain

**Keywords:** chimeric antigen receptor (CAR)T-cell, pediatric intensive care unit, cytokine release syndrome (CRS), immune effector cell associated neurotoxicity syndrome, acute lymphoblastic leukemia

## Abstract

**Introduction:**

Chimeric antigen receptor (CAR)T-cell CD19 therapy is an effective treatment for relapsed/refractory B-cell acute lymphoblastic leukemia. It can be associated with life-threatening toxicities which often require PICU admission. Purpose: to describe clinical characteristics, treatment and outcome of these patients.

**Methods:**

Prospective observational cohort study conducted in a tertiary pediatric hospital from 2016-2021. Children who received CAR-T admitted to PICU were included. We collected epidemiological, clinical characteristics, cytokine release syndrome (CRS) and immune effector cell-associated neurotoxicity syndrome (ICANS), treatment, length of stay and mortality.

**Results:**

CAR T-cells (4-1BB constructs) were infused in 59 patients. Twenty-four (40.7%) required PICU admission, length of stay was 4 days (IQR 3-6). Median age was 8.3 years (range 4-24). Patients admitted to PICU presented higher disease burden before infusion: 24% blasts in bone marrow (IQR 5-72) vs. 0 (0-6.9), p<0.001. No patients with <5% blasts were admitted to PICU. Main reasons for admissions were CRS (n=20, 83.3%) and ICANS (n=3, 12.5%). Fourteen patients (58.3%) required inotropic support, 14(58.3%) respiratory. Sixteen patients (66.6%) received tocilizumab, 10(41.6%) steroids, 6(25.0%) anakinra, and 5(20.8%) siltuximab. Ten patients (41.6%) presented neurotoxicity, six of them severe (ICANS 3-4). Two patients died at PICU (8.3%) because of refractory CRS-hemophagocytic lymphohistyocitosis (carHLH) syndrome. There were no significant differences in relapse rate after CAR-T in patients requiring PICU, it was more frequently CD19 negative (p=0.344).

**Discussion:**

PICU admission after CAR-T therapy was mainly due to CRS. Supportive treatment allowed effective management and high survival. Some patients presenting with carHLH, can suffer a fulminant course.

## 1 Introduction

Chimeric antigen receptor (CAR) T-cell therapy is an effective treatment for relapsed/refractory B-cell acute lymphoblastic leukemia (r/r B-ALL) (1, 2). In children with r/r ALL, anti-CD19 CAR T-cell therapy has shown high remission rates and overall survival rate around 75% at 12 months (2, 3).

After CAR T-cell infusion, these and other immune cell release cytokines which trigger a supraphysiologic inflammatory state. This can lead to severe toxicities, which can be life-threatening and associated with significant morbidity (1, 4, 5) and admission to Pediatric Intensive Care Unit (PICU): after CAR T cell therapy up to 47% of patients require PICU management (1, 6–10).

The most common nonhematologic adverse effects are cytokine release syndrome (CRS) and immune effector cell-associated neurotoxicity syndrome (ICANS) (4). CRS is observed in the majority of infused patients with ALL (85-93%), being severe in up to 48%. It presents with fever and constitutional symptoms but it can rapidly progress to circulatory shock, acute respiratory failure and organ dysfunction (1, 7, 11). Neurotoxicity usually occurs simultaneously with CRS or shortly after CRS resolution but sometimes independently of it (1, 4, 6, 12). Symptoms are diverse and include delirium, tremors, ataxia, aphasia, seizures and can progress to global encephalopathy and even cerebral edema in a small number of patients (1, 13). ICANS is observed in up to 64% of patients, being severe in 21% of cases (2, 8, 11). The incidence and severity of these complication differs significantly among different constructs, CD28 costimulatory domain is more frequently associated to severe neurotoxicity (14) compared to 4-1BB costimulatory domains. Following the recommendations by the European Bone Marrow Transplantation (EBMT) (15) and American Society of Transplant and Cell Therapy (ASTC) (9, 16, 17), ICU management should be considered for patients with grade 3-4 toxicities. In this situation, treatment with Tocilizumab (antiinterleukin-6 receptor (IL-6R)) and corticosteroids are recommended, as well as other support measures (fluid resuscitation, inotropic support, mechanical ventilation). For patients with ICANS 3-4, treatment with corticosteroids may be initiated, considering also anti-epileptics and treatment to reduce intracranial pressure in case of cerebral edema.

Azoulay et al. (18), in a multicentric study including adult patients showed 27% ICU admission and 90-day mortality of 22.4%. However, very little information is available on the management and outcomes of pediatric patients with B-ALL admitted to the PICU following CD19 CAR T-cell therapy.

Therefore, our main purpose was to describe the clinical characteristics, supportive treatment and clinical evolution of this specific population. The secondary objectives were to determine risk factors for PICU admission, and to compare the evolution of patients who required PICU admission with those who did not.

## 2 Materials and methods

A prospective observational cohort study was conducted in a tertiary pediatric hospital from April 2016 to December 2021. Eligible patients were children and young adults who received CAR T-cell therapy due to r/r B-ALL and required PICU admission (Flowchart of included patients available in **Supporting Information 1**).

Two different types of CAR19 4-1BB constructs were used: 1) tisagenlecleucel either in clinical trials (CTL019, EudraCT 2013-003205-25 and EudraCT 2016-001991-31) or after marketing authorization, and 2) ARI-0001 cells (varnimcabtagene autoleucel), an academic CAR19T-cell within a clinical trial (EudraCT 2016-002972-29) or on a compassionate use basis (19, 20). Exclusion criteria were parents’ refusal to participate in the study and patients receiving other CAR T-cell products. The study followed STROBE guidelines for cohort reports. Institutional review board approval was obtained from the local research institution and ethics committee in accordance with local ethics regulations (Study code PIC-85-21, approved on April 29th 2021, entitled “Epidemiological registration of pediatric and young adults between 1-25 years old treated with CAR T-cell therapy”). The study complies with the Declaration of Helsinki and was performed according to ethics committee approval. Written informed consent was obtained from all parents.

Different variables were collected from the electronic medical record: epidemiologic data, previous treatment, lymphodepleting chemotherapy, disease burden prior to lymphodepletion treatment, supportive treatment and complications in PICU, blood test results, PICU and hospital length of stay (LOS). PRISM III score (21) was calculated to determine the risk of mortality.

Primary outcome to compare patients who required PICU and those who did not was in-hospital mortality at day 30. Secondary outcomes were hospital LOS, and relapse (both, CD19 positive or negative).

Definitions and severity graduation: Relapsed leukemia was defined as the reemergence of measurable residual disease (MRD) by flow-cytometry (FC) or by morphology after achieving complete remission (CR). Patients with refractory leukemia did not reach MRD negative CR after ≥2 treatment lines. Disease burden was defined as the highest blast percentage measured in bone marrow by morphology and by FC after bridging chemotherapy. Length of stay (LOS) was defined as the duration of a single episode of hospitalization, from admission to discharge. In-hospital mortality was the number of patients who died while in the hospital.

Overall survival (OS) was calculated from the date of CAR T-cell infusion.

Inotropic support was quantified using the vasoactive-inotropic score (VIS) (22) as follow: VIS = dopamine dose (µg/kg/min) + dobutamine dose (µg/kg/min) + 100 x epinephrine dose (µg/kg/min) + 100 x norepinephrine dose (µg/kg/min) + 10 x milrinone dose (µg/kg/min) + 10,000 x vasopressin dose (U/kg/min).

Toxicity derived from CAR T-cell therapy was graded prospectively according to different scales depending on the clinical trial and the time period (16). In order to compare the results, we have retrospectively converted all the grades according to the ASTCT consensus guidelines (23).

The support strategy was as per the standard practice in PICU, based on the hemodynamic state and parameters of oxygenation (**Supporting Information 2**). An anti-interleukin-6R antibody (tocilizumab) was administered in case of hemodynamic instability. The indication of tocilizumab had changed over the time, with tocilizumab being used in the first clinical trial only in patients with severe CRS (grade 4) to progressively being used earlier in the course of the disease, after it was demonstrated that tocilizumab use did not have a deleterious impact on CAR19 expansion (24). If no clinical response was observed, a second dose was administered and/or steroids were initiated. In patients refractory to both treatments, other anti-cytokines (siltuximab, anakinra) were used. For patients with ICANS concomitantly with CRS, or isolated ICANS with no response to steroids, siltuximab was considered instead of tocilizumab.

In case of suspected infection, broad-spectrum antibiotics were initiated and diagnostic tests were made. Biomarkers were checked periodically.

Statistical analysis was performed using the SPSS25.0^®^ program. Categorical variables were expressed as frequency and percentage and continuous ones as median and interquartile range (IQR). Data were analyzed with non-parametric tests, using chi-square test to compare categorical variables and Mann-Whitney U test for continuous variables. Backward stepwise logistic regression was performed to analyze independent risk factors of the need for PICU admission, considering those variables that shown statistically significant differences in the univariate analysis. The relationship of continuous variables (biomarkers) with PICU admission was analyzed using ROC curves and the cut-offs points were determined using Youdens’s to obtain the best fit. A p-value<0.05 was considered significant.

## 3 Results

During the study, CAR-T cells were infused in 59 patients and 24 (40.7%) required PICU admission. Global median age of the study cohort at infusion was 9.0 years (IQR 6-13.7, range 1-25) and 44.1% (n=26) were female. Global hospital LOS was 16 days (IQR 14-24.8, range 4-84). **Table 1** describes clinical characteristics of the study cohort and compares patients regarding the need for PICU admission.

**Table 1:** General characteristics of the study cohort.

Variable	All patients n (%) (*n*=59)	No admission at PICU (*n*=35)	Admission at PICU (*n*=24)	p-value
Female, *n* (%)	26 (44.1)	14 (40)	12 (50)	0.45
Age, years (IQR)	9 (6-13.7)	9.4 (6.6-14.3)	8.3 (5.4-11)	0.66
Previous relapse, *n* (%)				
Refractory, *n* (%)	1 (1.7)	0 (0)	1 (4.2)	0.23
1st relapse, *n* (%)	12 (20.3)	5 (14.3)	7 (29.2)	
≥ 2nd relapse, *n* (%)	46 (77.9)	30 (85.7)	16 (66.7)	
Prior CNS involvement, *n* (%)	24 (40.7)	14 (40)	10 (41.7)	0.90
Previous HSCT, *n* (%)	34 (57.6)	24 (68.6)	10 (41.7)	0.04
Prior Radiotherapy, *n* (%)	17 (28.8)	10 (28.6)	7 (29.2)	0.93
Tumor burden (BM blasts by FC (%)*	5 (44)	0 (6.9)	24 (67)	<0.001

CNS, central nervous system; HSCT, Hematopoietic Stem Cell Transplant; FC, flow cytometry; RDT, Radiotherapy. *Blasts percentage after bridging chemotherapy.

### 3.1 PICU admission

Twelve of 24 patients (50%) admitted to the PICU were males. Median age at infusion was 8.3 years (IQR 5.9-11, range 4-24). Median disease burden (by FC) before lymphodepleting chemotherapy was 24% (IQR 5-72 range <0.01-98). Median time from CAR infusion to PICU admission was 4 days (IQR 3-6).

Patient’s characteristics and supportive treatment are included in **Table 2**. The main reasons for admission were CRS (n=20, 83.3%) and ICANS (n=3, 12.5%). One patient, who had a recent pulmonary infection, was admitted due to respiratory failure buy did not develop neither CRS nor neurotoxicity. The distribution of patients regarding the CRS and ICANS are described in **Table 2**. Regarding neurological symptoms, fourteen patients (58.4%) present any degree of neurological involvement. Only one patient (4.2%) presented ICANS 4 that required intubation and mechanical ventilation. None of them had cerebral edema and were no cases of ICANS 5.

**Table 2:** Clinical characteristics and supportive treatment of patients admitted to PICU.

	Admitted to PICU (n=24)
Female (%)	12 (50%)
Age	
*<5 years* *≥5 and <15 years* *≥15 and <25 years*	3 (12.5%) 16 (66.6%) 5 (20.9%)
PRISM score*	9 (IQR 3.75-10.5)
CART	
Tisagenlecleucel** ARI-0001***	19 (79.2%) 5 (20.8%)
CRS onset <24h from infusion	10/24 (41.7%)
CRS onset <72h from infusion	17/24 (70.8%)
CRS grade	
*Grade 1-2* *≥ Grade 3*	10 (41.7%) 13 (58.3%)
ICANS grade	
*Grade 1-2* *≥ Grade 3*	2 (8.3%) 6 (25.0%)
Inotropic support *VIS*	14 (58.3%) 15 (IQR 9-47)
Respiratory support *Conventional oxygen therapy* *High flow nasal cannula* *Non-invasive MV* *MV*	14 (58.3%) 6 (25.0%) 3 (12.5%) 1 (4.1%) 4 (16.7%)
CAR T-cell toxicities treatment	
*Tocilizumab* *Steroids* *Siltuximab* *Anakinra*	16 (66.6%) 10 (41.6%) 5 (20.8%) 6 (25.0%)
Renal replacement therapy	1 (4.1%)
ECMO	0 (0%)
LOS (days)	4 (IQR 2-7)
Mortality	2 (8.3%)

PRISM, Pediatric Risk of Mortality Score; CRS, cytokine release syndrome; ICANS, Immune effector cell-associated neurotoxicity syndrome; VIS, vasoactive-intropic score; MV, mechanical ventilation; ECMO, extra-corporeal membrane oxygenation; LOS, length of stay. *Data of two patients was not available. **Five patients were from the clinical trial and 14 received commercial CART. ***Two received single infusion and 2 fractionated dosing.

Patients who required PICU admission presented greater complications such as coagulopathy and infection as it is shown in **Supporting Information 3**. Regarding the coagulopathy, patients in PICU frequently had coagulation disorder (48.5%), being severe in 8 cases (33.3%) requiring plasma transfusion, fibrinogen (6 patients) and vitamin K. No massive bleeding occurred.

### 3.2 Support in PICU

Supportive treatment received during PICU admission is detailed in **Table 2**. The reason for establishing invasive mechanical ventilation was shock without response to medium doses of inotropic drugs in three cases and severe neurological impairment due to ICANS in one case. One patient needed renal replacement therapy due to multiorgan failure in the context of CRS grade 5-hemophagocytic lymphohistiocystosis syndrome (carHLH) and died.

Sixteen patients (66.6%) received tocilizumab, 8 (50.0%) with CRS 1-2 and 8 (50.0%) with CRS 3-4. This treatment was administered in the ward prior to PICU admission in 7 cases (43.8%). The median time from CAR-T infusion to tocilizumab administration was 4.5 days (IQR 2-7). In five cases, a second dose was administered, at a median of 2.5 days (IQR 1.8-6.3) after the first dose. Four of them were also treated with steroids.

Steroids were used in ten patients (41.6%), and 5 patients (20.8%) received siltuximab as a third line of therapy for severe CRS (after tocilizumab and steroids) or as second line for ICANS (after steroids). Anakinra was used in 6 patients (25.0%): 4 with severe CRS, 1 with refractory ICANS and 1 as prophylaxis for neurotoxicity. Two more patients received it as prophylaxis for neurotoxicity early in the ward, not requiring PICU admission after its administration.

Only one was admitted to PICU due to infection (Gram negative sepsis). However, 9/24 (37%) of the patients presented confirmed infections: 6 viral and 5 bacterial (2 patients had coinfection). Specific type of infection, symptoms and time onset are detailed in **Supporting Information 4**.

### 3.3 Blood test results

**Figure 1:**
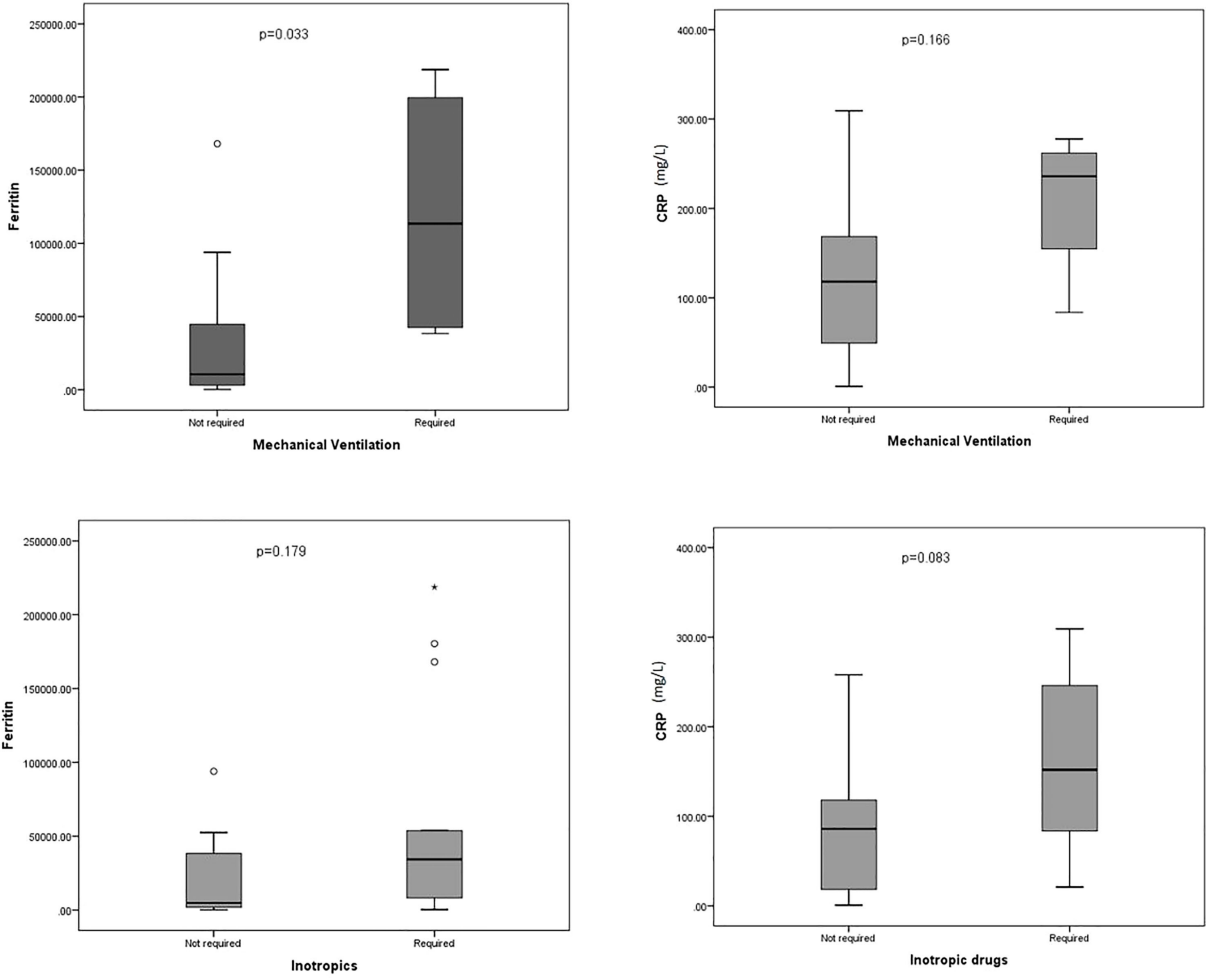
Box plot representing the maxim values for ferritin and C-reactive protein (CRP) in patients in PICU regarding the need for mechanical ventilation (superior) and inotropic support (inferior). *Outlier.

The median maximum value of C-reactive protein (CRP) and ferritin were 139 mg/dL (IQR 40.8-221.6) and 12023 ug/l (IQR 3372-52077), statistically significantly higher than those of patients who were not admitted to PICU: CRP 43.5 mg/dL (IQR 17.5-96.8) and ferritin, 2755 (IQR 1434-34842) with p<0.001 for ferritin and p=0.002 for CRP (**Supporting Information 5**). Those patients who required inotropic drugs showed higher values of ferritin, but no differences were observed for CRP (**Figure 1**). No statistically significant differences were observed in ferritin and CRP levels regarding the need for invasive mechanical ventilation. The two patients who died as a consequence of CRS grade 5-carHLH presented the highest ferritin (218,725 and 180,395 ug/l) and bilirubin levels (7 mg/dl for both patients), as well as tumor burden, but CRP did not show statistically significant differences, as it is shown in **Supporting Information 5**.

**Figure 2:**
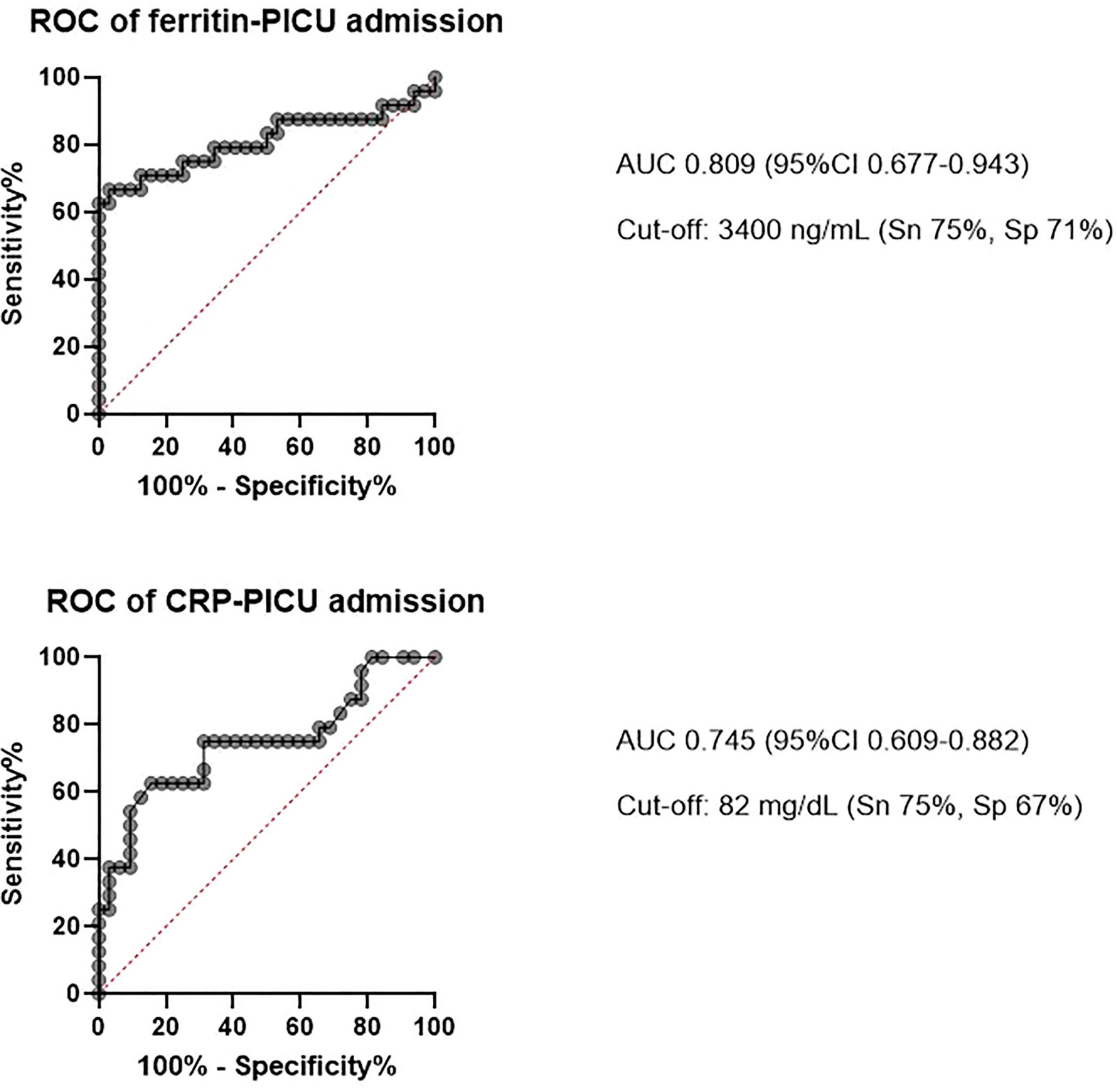
ROC curves representing the capacity of the biomarkers to predict the need for admission to the Pediatric intensive care unit (PICU).

Considering all the patients and the risk factors for needing admission to PICU, ROC curves analyses were performed to determine the capacity of ferritin and CRP to predict this outcome, as it is shown in **Figure 2**. The multivariate analysis (considering sex, HSCT, CRP and ferritin) showed independent association between CRP and ferritin and the need for PICU admission (OR 7.34 (95%CI 1.68-31.99) for CRP, 4.7 (95%CI 1.15-19.22) for ferritin), as it is shown in **Supporting Information 6**.

### 3.4 Clinical evolution of patients admitted to the PICU

PICU LOS was 4 days (IQR 2-7) and hospital LOS 29 days (IQR 16.5-39.5). Four patients presented carHLH, with two of them presenting a fulminant course, suffering refractory shock with severe lactic acidosis and dying day +4 and day +6 after infusion. The mortality rate in our series was 3.34% for all the patients treated with CAR-T and 8.3% among those admitted to PICU.

Outcomes comparison between patients admitted vs. non-admitted to PICU

The outcomes comparison between patients admitted to PICU and those who not are detailed in **Table 3**. No significant differences were observed regarding the evolution towards refractory/relapsed leukemia. CAR T-cell loss seems to be more frequent in patients not admitted to PICU (40% vs. 27%) but this difference did not reach statistical significance (p=0.252). Five patients received a second infusion due to CAR T-cell loss, but none of them required PICU admission. The type of relapse after CAR in patients who required PICU admission was predominately CD19 negative while the frequency of CD19+ and CD19- relapses were similar in those not requiring PICU admission. Mortality after PICU discharge was higher in patients who needed PICU admission (59 vs 17%, p=0.04, **Table 3**).

**Table 3:** Outcomes regarding the need for PICU admission.

	Total (n=57)*	PICU admission not required (n=35)	Need for PICU admission (n=22)*	p-value
Hospital LOS, days (IQR)	16 (14-24.8)	14 (13-16)	29 (16.5-39.5)	<0.001
Refractory leukemia, *n* (%)	3 (5.3)	0 (0)	3 (13.6)	0.365
CAR loss, *n* (%)	20 (35.1)	14 (40.0)	6 (27.3)	0.252
Relapse after CAR, *n* (%)	23 (40.3)	12 (34.3)	11 (50.0)	0.344
CD19+, *n* (%)	9 (39.1)	7 (58.3)	2 (18.2)	0.049
CD19-, *n* (%)	14 (60.9)	5 (41.7)	9 (81.8)	
Mortality in evolution *n* (%)	19 (33.3)	6 (17.1)	13 (59.1)	0.004

*Excluded 2 patients who died in PICU. CAR, chimeric antigen receptor; LOS, length of stay; PICU, pediatric intensive care unit.

## 4 Discussion

The current study provides a comprehensive analysis of pediatric patients and young adults with ALL admitted to PICU after CAR T-cell therapy. The major findings in our study were: 1)PICU admission after CAR-T therapy in pediatric patients with ALL is frequent, mainly due to CRS; 2)as previously described, high tumor burden is a risk factor for severe CRS and ICANS and, therefore, PICU admission; 3)supportive treatment allowed effective management of toxicities with high survival; 4)relapse was more frequently CD19 negative among patients admitted to PICU; 5)unfavorable outcome after PICU discharge was more frequent among patients requiring intensive care management (59% vs. 17%).

Our incidence of patients requiring ICU admission is in concordance with previous studies reporting 45-50% ICU admission rate after CAR T-cell therapy in patients with ALL (1, 25, 26). CRS was the most frequent nonhematologic adverse effect (78%), and severe CRS was present in 22% of the patients, in line with other reported series (16-48%) (11). Of note, around 40% of patients presented infection after CAR T-cell therapy, as reported in other studies focused in similar population (27).

As previously described (28, 29), high disease burden was significantly associated with PICU admission. Notably, no patient with MRD (<5% blasts) required PICU admission, so perhaps these patients with low disease burden could receive CAR T-cells in an outpatient setting.

The second most frequent reason for admission was neurotoxicity; even though only 13% of patients were admitted due to neurotoxicity, one third had neurological symptoms during their PICU stay, one of them ICANS grade 4. Our results are consistent with previous studies reporting ≈10% of severe ICANS in patients treated with tisagenlecleucel (2, 3). Most patients suffering severe ICANS developed this complication after severe CRS (30).

All included patients received CAR T-cell with costimulatory domain 4-1BB and the majority of PICU admitted patients received tisagenlecleucel. This is important to take into account when considering incidence and severity of complications as the toxicity among products differ, particularly in neurotoxicity, in which products with CD28 costimulatory are more frequently associated with ICANS (up to 67%, severe 20-30%) and even cerebral edema which can be fatal (2, 14, 31).

Regarding supportive treatment, approximately 60% of patients admitted to the PICU required inotropic support and 20% mechanical ventilation. Previous studies focused in patients with hematologic malignancies without history of CAR T-cell nor allogeneic hematopoietic stem cell transplantation (HSCT) showed that 30-59% patients needed ionotropic support and 24-59% mechanical ventilation (32–34), which suggest that all hematologic patients are a high-risk population probably due to their immunosuppression and treatment-related toxicities.

In our institution, CRS management has changed over time. According to the initial guidelines the use of tocilizumab was recommended only in patients with severe CRS grade 3-4 after fluid boluses and vasopressor treatment (35–37). Several factors contributed to the change in CRS management and tocilizumab indications. First, earlier use of vasopressors to avoid multiple fluid boluses in case of hypotension. This, in our experience, improved the hemodynamic and respiratory status of the patients by reducing the fluid overload and pulmonary edema in the setting of vascular leakage and endothelial damage caused by CRS. Second, by using earlier tocilizumab in CRS grade 2 or even in grade 1 if the patient had a high tumor burden and risk of severe CRS. This was supported by several studies that showed that the use of tocilizumab did not affect CAR T-cell efficacy (2, 8, 26, 38). Furthermore, it was demonstrated that the use preemptive tocilizumab in selected patients with high risk of severe CRS decreased the expected incidence of grade 4 (24) CRS. In fact, current guidelines recommend the early use of tocilizumab to reduce the rate of severe CRS (4, 17). In addition to tocilizumab, which is now approved for the treatment of CAR T-related CRS, we have used other monoclonal antibodies off-label in cases of refractory CRS or severe ICANS. For patients with ICANS concomitantly with CRS, or isolated ICANS with no response to steroids, siltuximab was considered instead of tocilizumab due to its mechanism of action, binding directly IL-6 and reducing the IL-6 crossing the blood-brain barrier (5, 31). Anakinra, IL-1 receptor inhibitor, is increasingly used to treat severe CRS and ICANS unresponsive to front-line therapy (39, 40) Preclinical data suggest that IL-1 contributes to CRS and ICANS1 and that its blockade may be effective in the prevention and treatment of CRS/ICANS without affecting CAR T-cell T efficacy (39–41). In our institution, anakinra was used both for treatment (2 mg/kg every 8 hours, maximum 10 mg/8h) and prophylaxis (2 mg/kg every 24 hours). As a treatment, it was started in patients with severe CRS or with an important hemophagocytic component, as well as in those patients with ICANS. As a prophylaxis, it was started in patients with high risk of neurotoxicity due to CNS infiltration or neurologic comorbidities prior to CAR T-cell treatment.

The characterization of those patients who are at high risk of severe adverse effects is crucial to optimize their support and ensure the adequacy of treatment. Several biomarkers, such as C reactive protein and ferritin, have been proposed as CRS predictors (42). In our cohort, high ferritin levels were present in severe patients with inotropic support.

The mortality rate of 8.7% is similar to multicentric studies in adult and pediatric patients admitted to ICU after CAR T-cell therapy (5.8% in adults with lymphoma/ALL and 7% in children with ALL) (18). In our series, two patients died as a consequence of fulminant CRS5-carHLH. CAR T-cell-associated severe carHLH is a rare toxicity related to CAR T-cell therapy which occurs in 1-14% of patients, according to different series (37, 43). Its true incidence is unknown due to clinical overlap with high-grade CRS and the high variability in diagnostic criteria used over the last years. It is characterized by hyperinflammatory syndrome hyperferritinaemia, multiorgan disfunction and/or evidence of haemophagocytosis in the bone marrow, and its onset usually occur after initial improvement of the CRS. The two patients included in our series fulfilled most of laboratory criteria according to new carHLH definition, but presented a fulminant course without improvement of CRS before multiorgan failure occurred (44). However, cytokine profiles of CRS and carHLH are similar, which could be explained by a continued evolution of the inflammatory process. The initial management of carHLH is the same that patients with grade ≥3CRS. Etoposide, anakinra, cyclophosphamide and extracorporeal cytokine adsorption have been reported with success in few cases (44, 45), while other treatments such as ruxolitinib have been recently described. Whereas patients with CRS usually respond to initial therapy, carHLH is associated with high mortality (43). Suspicion of carHLH should be raised when early occurrence (<5 days after infusion) of ferritin peak (>10,000 ng/mL) or early organ dysfunction and hemophagocytosis in BM (43, 46).

In our cohort, relapse was more frequent in the patients admitted to PICU (50 vs 34%) but this difference was not significant. Nevertheless, type of relapse was different: the majority of relapses in patients admitted to PICU admission were CD19- (82%). These might probably relate to high disease burden, which has been associated with higher risk of CD19- relapse (47). However, it is important to take into consideration that other factors, which are not included in this analysis, have been related to CD19- relapses, such as prior bilatumomab nonresponse or older age (48–50).

Our study has some limitations. Even though it is a relatively large series of this specific population, the sample size is small and this could influence the results and limits the identification of risk factors for mortality. Also, since CAR T-cell is a relatively novel therapy, guidelines for diagnostic and management and treatment decisions changed over the study period.

In conclusion, critical care management has become an essential part of CAR T-cell therapy. Further research regarding risk factors associated to PICU admission and mortality, as well as specific intensive care guidelines, are crucial to optimize their support and ensure the most effective treatment.

## Data availability statement

The raw data supporting the conclusions of this article will be made available by the authors, without undue reservation.

## Ethics statement

The studies involving human participants were reviewed and approved by Comité de Ética de Investigación con medicamentos, Hospital Sant Joan de Déu, Barcelona (Spain). Written informed consent to participate in this study was provided by the participants’ legal guardian/next of kin.

## Author contributions

All authors contributed to the study conception and design. Material preparation, data collection and analysis were performed by MC-B, AA-S, SB-P, CR. The first draft of the manuscript was written by MC-B and SB-P and all authors commented on previous versions of the manuscript. All authors contributed to the article and approved the submitted version.

## References

[1] Maude SL Frey N Shaw PA Aplenc R Barrett DM Bunin NJ. Chimeric antigen receptor T cells for sustained remissions in leukemia. N Engl J Med (2014) 371(16):1507–17. doi: 10.1056/NEJMoa1407222 PMC4267531 25317870

[2] Maude SL Laetsch TW Buechner J Rives S Boyer M Bittencourt H. Tisagenlecleucel in children and young adults with B-cell lymphoblastic leukemia. N Engl J Med (2018) 378(5):439–48. doi: 10.1056/nejmoa1709866 PMC5996391 29385370

[3] Pasquini MC Hu ZH Curran K Laetsch T Locke F Rouce R. Real-world evidence of tisagenlecleucel for pediatric acute lymphoblastic leukemia and non-Hodgkin lymphoma. Blood Adv (2020) 4(21):5414–24. doi: 10.1182/bloodadvances.2020003092 PMC7656920 33147337

[4] Schubert ML Schmitt M Wang L Ramos CA Jordan K Müller-Tidow C. Side-effect management of chimeric antigen receptor (CAR) T-cell therapy. Ann Oncol (2021) 32(1):34–48. doi: 10.1016/j.annonc.2020.10.478 33098993

[5] Brudno JN Kochenderfer JN Transplantation E Branch I States U. Recent advances in CAR T-cell toxicity: Mechanisms, manifestations and management. Blood Re (2019) 34:45–55. doi: 10.1016/j.blre.2018.11.002 PMC6628697 30528964

[6] Schuster SJ Svoboda J Chong EA Nasta SD Mato AR Anak Ö. Chimeric antigen receptor T cells in refractory B-cell lymphomas. N Engl J Med (2017) 377(26):2545–54. doi: 10.1056/nejmoa1708566 PMC5788566 29226764

[7] Porter D Frey N Wood PA Weng Y Grupp SA. Grading of cytokine release syndrome associated with the CAR T cell therapy tisagenlecleucel. J Hematol Oncol (2018) 11(1):1–12. doi: 10.1186/s13045-018-0571-y 29499750 PMC5833070

[8] Park JH Rivière I Gonen M Wang X Sénéchal B Curran KJ. Long-term follow-up of CD19 CAR therapy in acute lymphoblastic leukemia. N Engl J Med (2018) 378(5):449–59. doi: 10.1056/nejmoa1709919 PMC6637939 29385376

[9] Neelapu SS. Managing the toxicities of CAR T-cell therapy. Hematol Oncol (2019) 37(S1):48–52. doi: 10.1002/hon.2595 31187535

[10] Myers RM Fitzgerald JC DiNofia A Wray L Leahy AB Li Y. Inpatient and intensive care unit resource utilization after CD19-targeted Chimeric Antigen Receptor T-cell Therapy (CART19) for pediatric acute lymphoblastic leukemia (ALL). Biol Blood Marrow Transplant (2020) 26(3):S202–3. doi: 10.1016/j.bbmt.2019.12.695

[11] Gardner RA Finney O Annesley C Brakke H Summers C Leger K. Intent-to-treat leukemia remission by CD19 CAR T cells of defined formulation and dose in children and young adults. Blood (2017) 129(25):3322–31. doi: 10.1182/blood-2017-02-769208 PMC5482103 28408462

[12] Lee DW Gardner R Porter DL Louis CU Ahmed N Jensen M. Current concepts in the diagnosis and management of cytokine release syndrome. Blood (2014) 124(2):188–95. doi: 10.1182/blood-2014-05-552729 PMC4093680 24876563

[13] Torre M Solomon IH Sutherland CL Nikiforow S DeAngelo DJ Stone RM. Neuropathology of a case with fatal CAR T-cell-associated cerebral edema. J Neuropathol Exp Neurol (2018) 77(10):877–82. doi: 10.1093/jnen/nly064 30060228

[14] Cappell KM Kochenderfer JN. A comparison of chimeric antigen receptors containing CD28 versus 4-1BB costimulatory domains. Nat Rev Clin Oncol (2021) 18(11):715–27. doi: 10.1038/s41571-021-00530-z 34230645

[15] Ayuketang FA Jäger U. Management of Cytokine Release Syndrome (CRS) and HLH. In: Kröger N Gribben J Chabannon C Yakoub-Agha I Einsele H, editors. The EBMT/EHA CAR-T Cell Handbook. Cham (CH): Springer (2022). Chapter 26. 36122067

[16] Lee DW Santomasso BD Locke FL Ghobadi A Turtle CJ Brudno JN. ASTCT consensus grading for cytokine release syndrome and neurologic toxicity associated with immune effector cells. Biol Blood Marrow Transplant (2019) 25(4):625–38. doi: 10.1016/j.bbmt.2018.12.758 PMC12180426 30592986

[17] Yakoub-Agha I Chabannon C Bader P Basak GW Bonig H Ciceri F. Management of adults and children undergoing chimeric antigen receptor T-cell therapy: best practice recommendations of the European Society for Blood and Marrow Transplantation (EBMT) and the Joint Accreditation Committee of ISCT and EBMT (JACIE). Haematologica (2020) 105(2):297–316. doi: 10.3324/haematol.2019.229781 31753925 PMC7012497

[18] Azoulay É Castro P Maamar A Metaxa V de Moraes AG Voigt L. Outcomes in patients treated with chimeric antigen receptor T-cell therapy who were admitted to intensive care (CARTTAS): an international, multicentre, observational cohort study. Lancet Haematol (2021) 8(5):e355–64. doi: 10.1016/S2352-3026(21)00060-0 33894170

[19] Ortíz-Maldonado V Rives S Castellà M Alonso-Saladrigues A Benítez-Ribas D Caballero-Baños M. CART19-BE-01: A multicenter trial of ARI-0001 cell therapy in patients with CD19+ Relapsed/refractory malignancies. Mol Ther (2021) 29(2):636–44. doi: 10.1016/j.ymthe.2020.09.027 PMC7854276 33010231

[20] Ortiz-Maldonado V Rives S Español-Rego M Alonso-Saladrigues A Montoro M Magnano L. Factors associated with the clinical outcome of patients with relapsed/refractory CD19 + acute lymphoblastic leukemia treated with ARI-0001 CART19-cell therapy. J Immunother Cancer (2021) 9(12):1–4. doi: 10.1136/jitc-2021-003644 PMC8671976 34907029

[21] Pollack MM Ruttimann UE Getson PR. Pediatric risk of mortality (PRISM) score. Crit Care Med (1996) 24(5):743–52. doi: 10.1097/00003246-199605000-00004 3048900

[22] McIntosh AM Tong S Deakyne SJ Davidson JA Scott HF. Validation of the vasoactive-inotropic score in pediatric sepsis. Pediatr Crit Care Med (2017) 18(8):750–7. doi: 10.1097/PCC.0000000000001191 PMC5548505 28486385

[23] Pennisi M Jain T Santomasso BD Mead E Wudhikarn K Silverberg ML. Comparing CAR T-cell toxicity grading systems: Application of the ASTCT grading system and implications for management. Blood Adv (2020) 4(4):676–86. doi: 10.1182/bloodadvances.2019000952 PMC7042979 32084260

[24] Kadauke S Myers RM Li Y Aplenc R Baniewicz D Barrett DM. Risk-adapted preemptive tocilizumab to prevent severe cytokine release syndrome after CTL019 for pediatric B-cell acute lymphoblastic leukemia: A prospective clinical trial. J Clin Oncol (2021) 39(8):920–30. doi: 10.1182/bloodadvances.2019000952 PMC8462622 33417474

[25] Fitzgerald JC Weiss SL Maude SL Barrett DM Lacey SF Melenhorst JJ. Cytokine release syndrome after chimeric antigen receptor T cell therapy for acute lymphoblastic leukemia. Crit Care Med (2018) 45(2):1–15. doi: 10.1097/CCM.0000000000002053 PMC5452983 27632680

[26] Neelapu SS Locke FL Bartlett NL Lekakis LJ Miklos DB Jacobson CA. Axicabtagene ciloleucel CAR T-cell therapy in refractory large B-cell lymphoma. N Engl J Med (2017) 377(26):2531–44. doi: 10.1056/nejmoa1707447 PMC5882485 29226797

[27] Maron GM Hijano DR Epperly R Su Y Tang L Hayden RT. Infectious complications in pediatric, adolescent and young adult patients undergoing CD19-CAR T cell therapy. Front Oncol (2022) 12:845540(March). doi: 10.3389/fonc.2022.845540 35356197 PMC8959860

[28] Hay KA Hanafi LA Li D Gust J Liles WC Wurfel MM. Kinetics and biomarkers of severe cytokine release syndrome after CD19 chimeric antigen receptor–modified T-cell therapy. Blood (2017) 130(21):2295–306. doi: 10.1182/BLOOD-2017-06-793141 PMC5701525 28924019

[29] Murthy H Iqbal M Chavez JC Kharfan-Dabaja M. Cytokine release syndrome: current perspectives. ImmunoTargets Ther (2019) 8:43–52. doi: 10.2147/ITT.S202015 31754614 PMC6825470

[30] Grant SJ Grimshaw AA Silberstein J Murdaugh D Wildes TM Rosko AE. Clinical presentation, risk factors, and outcomes of immune effector cell-associated neurotoxicity syndrome following chimeric antigen receptor T cell therapy: A systematic review. Transplant Cell Ther (2022) 28(6):294–302. doi: 10.1016/J.JTCT.2022.03.006 35288347 PMC9197870

[31] Wayne AS Huynh V Hijiya N Rouce RH Brown PA Krueger J. Three-year results from phase 1 of ZUMA-4: KTE-X19 in pediatric relapsed/refractory acute lymphoblastic leukemia. Haematologica (2023) 108(3):747–60. doi: 10.3324/haematol.2022.280678 PMC9973494 36263840

[32] Pillon M Sperotto F Zattarin E Cattelan M Carraro E Contin AE. Predictors of mortality after admission to pediatric intensive care unit in oncohematologic patients without history of hematopoietic stem cell transplantation: A single-center experience. Pediatr Blood Cancer (2019) 66(10):1–9. doi: 10.1002/pbc.27892 31250548

[33] Caballero M Faura A Margarit A Bobillo-Perez S Català A Alonso-Saladrigues A. Outcomes for paediatric acute leukaemia patients admitted to the paediatric intensive care unit. Eur J Pediatr (2021) 181(3):1037–45. doi: 10.1007/s00431-021-04292-9 34694507

[34] Ragoonanan D Bhar S Mohan G Beltramo F Khazal SJ Hurley C. A multicenter study of ICU resource utilization in pediatric , adolescent and young adult patients post CAR-T therapy. Front Oncol (2022) 12:1022901. doi: 10.3389/fonc.2022.1022901 PMC9638171 36353531

[35] Mahadeo KM Khazal SJ Abdel-Azim H Fitzgerald JC Taraseviciute A Bollard CM. Management guidelines for paediatric patients receiving chimeric antigen receptor T cell therapy. Nat Rev Clin Oncol (2019) 16(1):45–63. doi: 10.3324/haematol.2019.229781 30082906 PMC7096894

[36] Shimabukuro-Vornhagen A Gödel P Subklewe M Stemmler HJ Schlößer HA Schlaak M. Cytokine release syndrome. J Immunother Cancer (2018) 6(1):56. doi: 10.1186/s40425-018-0343-9 PMC6003181 29907163

[37] Neelapu SS Tummala S Kebriaei P Wierda W Gutierrez C Locke FL. Chimeric antigen receptor T-cell therapy-assessment and management of toxicities. Nat Rev Clin Oncol (2018) 15(1):47–62. doi: 10.1038/nrclinonc.2017.148 28925994 PMC6733403

[38] Davila ML Riviere I Wang X Bartido S Park J Chung SS. Efficacy and toxicity management of 19-28z CAR T cell therapy. Sci Transl Med (2014) 6(224):224–5. doi: 10.1126/scitranslmed.3008226 PMC4684949 24553386

[39] Lee DW Shah N. Chimeric antigen receptor T-cell therapies for cancer: a practical guide. Lee DW, Shah NN, editors. (Amsterdam, Netherlands: Elsevier) (2020). pp. 45–26.

[40] Diorio C Vatsayan A Talleur AC Annesley C Jaroscak JJ Shalabi H. Anakinra utilization in refractory pediatric CAR T-cell associated toxicities. Blood Adv (2022) 6(11):3398–403. doi: 10.1182/bloodadvances.2022006983 PMC9198909 35395068

[41] Park JH Sauter CS Palomba ML Shah GL Dahi PB Lin RJ. A phase II study of prophylactic anakinra to prevent CRS and neurotoxicity in patients receiving CD19 CAR T cell therapy for relapsed or refractory lymphoma. Blood (2021) 138(Supplement 1):96–6. doi: 10.1182/bloodadvances.2022006983

[42] Teachey DT Lacey SF Shaw PA Melenhorst JJ Maude SL Frey N. Identification of predictive biomarkers for cytokine release syndrome after chimeric antigen receptor T-cell therapy for acute lymphoblastic leukemia. Cancer Discov (2016) 6(6):664–79. doi: 10.1158/2159-8290 PMC5448406 27076371

[43] Logan GE Miller K Kohler ME Loi M Maddux AB. Outcomes of critically ill children with acute syndrome due to chimeric antigen receptor. Pediatr Crit Care Med (2022) 23(12):e595-e600. doi: 110.1097/PCC.0000000000003079 36194016 10.1097/PCC.0000000000003079 PMC9722524

[44] Martín-Rojas RM Gómez-Centurión I Bailén R Bastos M Diaz-Crespo F Carbonell D. Hemophagocytic lymphohistiocytosis/macrophage activation syndrome (HLH/MAS) following treatment with tisagenlecleucel. Clin Case Rep (2022) 10(1):1–6. doi: 10.1002/ccr3.5209 PMC8741874 35028140

[45] Hines MR Knight TE McNerney KO Leick MB Jain T Ahmed S. Immune effector cell associated hemophagocytic lymphohistiocytosis-like syndrome (IEC-HS). Transplant Cell Ther (2023), 1–16. doi: 10.1016/j.jtct.2023.03.006 36906275 PMC10330221

[46] Hines MR Keenan C Maron Alfaro G Cheng C Zhou Y Sharma A. Hemophagocytic lymphohistiocytosis-like toxicity (carHLH) after CD19-specific CAR T-cell therapy. Br J Haematol (2021) 194(4):701–7. doi: 10.1111/bjh.17662 PMC8756350 34263927

[47] Peterlin P Garnier A Le Bourgeois A Jullien M Seguin A Eveillard M. Dramatic Recovery after Etoposide Phosphate Infusion for Hemophagocytic Lymphohistiocytosis/Macrophage Activation Syndrome following Treatment with Tisagenlecleucel in a Young Patient with Relapsed Acute Lymphoblastic Leukemia: A Case Report. Acta Haematol (2022) 145(5):537–41. doi: 10.1159/000525576 35724631

[48] Dourthe ME Rabian F Yakouben K Chevillon F Cabannes-Hamy A Méchinaud F. Determinants of CD19-positive vs CD19-negative relapse after tisagenlecleucel for B-cell acute lymphoblastic leukemia. Leukemia (2021) 35(12):3383–93. doi: 10.1038/s41375-021-01281-7 34002027

[49] Lamble AJ Myers RM Taraseviciute A John S Yates B Steinberg SM. Preinfusion factors impacting relapse immunophenotype following CD19 CAR T cells. Blood Adv (2023) 7(4):575–85. doi: 10.1182/bloodadvances.2022007423 PMC9979750 35482927

[50] Myers RM Taraseviciute A Steinberg SM Lamble AJ Sheppard J Yates B. Blinatumomab nonresponse and high-disease burden are associated with inferior outcomes after CD19-CAR for B-ALL. J Clin Oncol (2021) 40:932–44. doi: 10.1200/JCO.21.01405 PMC8937010 34767461

