# Supplementary material for: Risk factors and outcome of Chimeric Antigen Receptor T-Cell patients admitted to Pediatric Intensive Care Unit: CART-PICU study
Source: Front Immunol. 2023 Aug 2;14:1219289. doi: 10.3389/fimmu.2023.1219289 (PMC10433898; doi:10.3389/fimmu.2023.1219289)
Supplement: Supplementary file 1 [file DataSheet_1.docx]

Supplementary Material

Risk factors and outcome of Chimeric Antigen Receptor T-Cell patients admitted to Pediatric Intensive Care Unit: CART-PICU study

**Caballero-Bellón, Marina MD^1^; Alonso-Saladrigues, Anna MD^1^; Bobillo-Perez, Sara PhD*^2,3^; Faura, Anna MD^1^; Arqués, Laura^1^; Rivera, Cristina MD^1^; Català, Albert PhD ^1,4^; Dapena, Jose Luis MD^1^; Rives, Susana PhD^1,4^ † and Jordan, Iolanda PhD^2,5^** †

*** Correspondence:** Corresponding Author: Sara Bobillo-Perez. E-mail: sbobillo@sjdhospitalbarcelona.org

# Supplementary Data

**1.1**  **Supplementary Data 1: Flowchart of patients admitted to the study**

* Manufacturing failure in 1 patients, second leukapheresis was performed

69 patients eligible for CAR T-cell therapy

*April 2016 – December 2021*

66 patients underwent leukapheresis

*2 patients died of disease progression*

*1 patient pending leukapheresis*

58 patients received CAR T-cell infusion

*1 patient pending infusion*

*7 patients were not infused and died of disease progression (3 of them were manufacturing failure)*

24 patients admitted to PICU after CAR T-cell infusion

**1.2 Supplementary Data 2: CRS and ICANS management in PICU**

**CRS ≥2**

**ICANS ≥3**

Fluid expansion

*Tachycardia +/- hypotension*

*Hypotension persistence*

Tocilizumab

Vasopressors

*Norepinephrine* (if no cardiac dysfunction)

Tocilizumab: 2^nd^ dose

+/- Steroids

*Refractory to treatment*

Siltuximab and anakinra

Steroids

*Refractory to treatment/*

*Concomitant CRS*

Siltuximab

**Supportive treatment**

- Continuous monitoring of vital signs
- Respiratory support according to oxygenation parameters and clinical condition: high-flow nasal cannula, non-invasive ventilation and invasive mechanical ventilation
- Broad-spectrum antibiotic if infection suspected: adjusted according to patient’s previous colonization or bacteria isolates in case of confirmed bacterial infection
- Anti-epileptic treatment if seizures

## Supplementary Data 3: Complications after infusion compared by the need for PICU admission

|  | **Total (n=59)** | **PICU admission not required (n=35)** | **Need for PICU admission (n=24)** | **p-value** |
| --- | --- | --- | --- | --- |
| TLS, *n* (%) | 1 (1.7) | 0 (0) | 1 (4.2) | 0.421 |
| Coagulation disorder, *n* (%) | 14 (23.7) | 3 (8.3) | 11 (45.8) | <0.001 |
| CRS, *n* (%) | 46 (78.0) | 23 (65.7) | 23 (95.8) | 0.005 |
| 1 | 26 (44.1) | 22 (66.7) | 4 (16.7) |  |
| 2 | 7 (11.9) | 1 (3) | 6 (25) |  |
| 3 | 6 (10.2) | 0 (0) | 6 (25) |  |
| 4 | 5 (8.5) | 0 (0) | 5 (20.8) |  |
| 5 | 2 (3.4) | 0 (0) | 2 (8.3) |  |
| Neurotoxicity, *n* (%) | 21 (35.6) | 5 (15.2) | 16 (66.7) | <0.001 |
| ICANS 0 | 47 (79.7) | 33 (100) | 10 (41.6) |  |
| ICANS 1 | 2 (3.4) | 0 (0) | 7 (30) |  |
| ICANS 2 | 2 (3.4) | 0 (0) | 2 (8.3) |  |
| ICANS 3 | 4 (6.8) | 0 (0) | 4 (16.7) |  |
| ICANS 4 | 2 (3.4) | 0 (0) | 1 (4.2) |  |
| Infection, *n* (%) | 13 (22) | 4 (11) | 9 (37) | 0.009 |
| Mortality, *n* (%) | 2 (3.4) | 0 (0) | 2 (8.3) | 0.161 |

CRS: cytokine release syndrome; ICANS: Immune effector cell-associated neurotoxicity syndrome; PICU: pediatric intensive care unit; TLS: Tumor lysis syndrome

**1.4 Supplementary Data 4: Infections of patients admitted to PICU**

|  | CRS onset | CRS grade | Symptoms | Infection onset | Infection |
| --- | --- | --- | --- | --- | --- |
| Patient 1 | D+18* | 2 | Skin lesion | D+12 | Herpes simplex |
|  |  |  | Fever | D+42 | *S haemolyticus* (bloodstream infection) |
| Patient 2 | D+6 | 2 | Diarrhea | D+6 | *C. Difficile* |
| Patient 3 | D+2 | 1 | Diarrhea | D+2 | *C. Difficile* |
| Patient 4 | D+2 | 3 | Upper respiratory symptoms | D+8 | Rhinovirus |
| Patient 5 | D+3 | 2 | Sepsis | D+19 | *E. Coli* (bloodstream infection) |
| Patient 6 | D+2 | 3 | Neurologic symptoms (memory loss) | D+30 | VHH-6 encephalitis |
|  |  |  | Asymptomatic | D+42 | Adenovirus infection |
|  |  |  | Diarrhea | D+30 | *C. difficile,* ADV and sapovirus |
| Patient 7 | D+1 | 3 | Cough | D+3 | Rhinovirus |
| Patient 8 | D+1 | 3 | Asymptomatic | D+19 | CMV reactivation |
| Patient 9 | D+1 | 4 | Fever | D+2 | *S. hominis* (bloodstream infection) |

* D+18 from 1st aliquot and D+11 from 3^rd^ aliquot

## 1.5 Supplementary Data 5
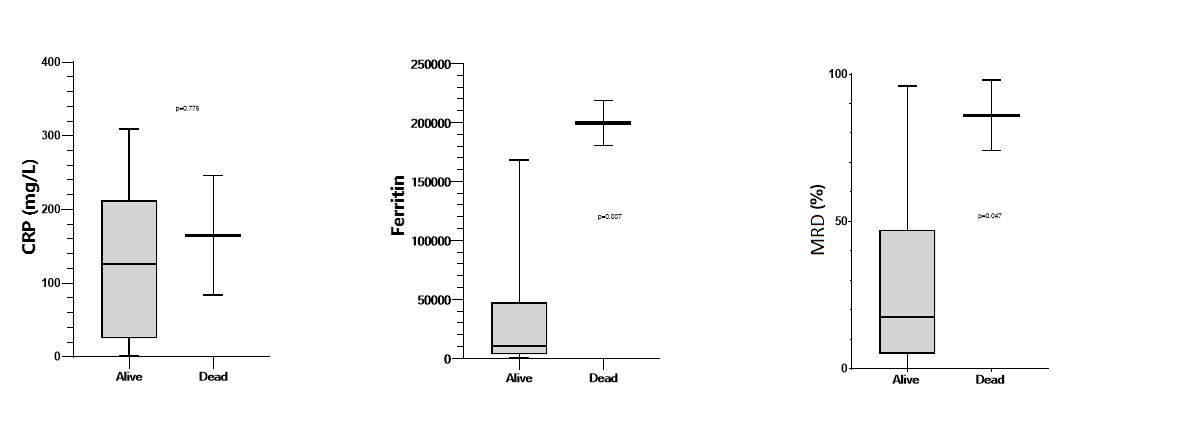


Box plot representing the maxim values for ferritin and C-reactive protein (CRP) and the minimal residual disease (MRD) comparing alive vs. death patients.


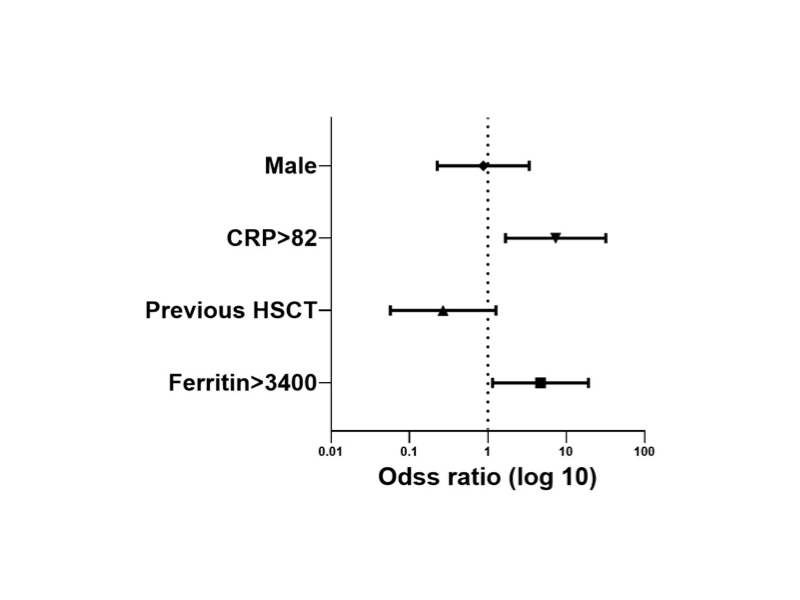
**1.6 Supplementary Data 6**

Forest plot representing the multivariate analysis considering the need for the admission to the pediatric intensive care unit (PICU) as the dependent factor. CRP: C- reactive protein; HSCT: hematopoietic stem cell transplant.
